# Retro MTA and tricalcium phosphate/retro MTA for guided tissue regeneration of periodontal dehiscence defects in a dog model: a pilot study

**DOI:** 10.1186/s40824-019-0163-0

**Published:** 2019-08-28

**Authors:** Omid Fakheran, Reza Birang, Patrick R. Schmidlin, Sayed Mohammad Razavi, Parichehr Behfarnia

**Affiliations:** 1 Department of Periodontics and Dental Research Center, School of Dentistry, Isfahan University of Medical Sciences, Isfahan, Iran; 2 Clinic of Conservative and Preventive Dentistry, Centre of Dental and Oral Medicine, University of Zurich, Plattenstrasse 11, 8032 Zurich, Switzerland; 3 Dental Implant Research Center and Department of Oral and Maxillofacial Pathology, School of Dentistry, Isfahan University of Medical Sciences, Isfahan, Iran

**Keywords:** Calcium silicate cement, MTA, Guided tissue regeneration, Bone regeneration

## Abstract

**Objectives:**

Retro MTA is a fast setting Calcium silicate cement used in endodontic regeneration procedures in recent years. Beta-tricalcium phosphate (β-TCP) is another common biomaterial used for bone augmentation procedures. The present pilot study was undertaken to evaluate and compare the efficacy of Retro MTA and a mixture of Retro MTA / β-TCP for periodontal tissue regeneration.

**Materials and methods:**

In 4 beagle dogs, periodontal dehiscence type defects were created. In each side, one dehiscence defect was left empty as a control site and three treatment modalities were randomly applied for the others: Retro MTA covered with a collagen membrane, Retro MTA + β-TCP covered with a membrane and covering the defect with a membrane without any bone augmentation. After 8 weeks Animals were sacrificed and Histomorphometric and histologic analysis were conducted.

**Results:**

Histologic analysis showed more cementum formation for both Retro MTA+ β-TCP (3.74 ± 0.34 mm) and Retro MTA group (3.24 ± 0.56 mm) compared to control group 1 (1. 15 ± 0.45 mm) and control group 2 (0.78 ± 0.65 mm). Formation of newly formed bone and cementum in the experimental groups were significantly higher as compared to the control groups (*P* < 0.0001).

**Conclusions:**

Retro MTA or Retro MTA+ β-TCP covered with a collagen membrane resulted in regeneration of periodontal tissues. However, Retro MTA+ β-TCP showed tendency towards better results than the use of Retro MTA alone.

## Introduction

Periodontal regeneration as defined by the American Academy of Periodontology is reproduction or reconstitution of lost or injured periodontal tissue [1]. Different treatment materials and techniques such as the use of resorbable or non-resorbable membranes, alone or in combination with various bone graft materials, growth factors or the application of enamel matrix derivative have been used in the context of periodontal regeneration with varying degrees of success [2–4].

Calcium silicate cements (CSCs) such as mineral trioxide aggregate (MTA) were introduced in dental practice for the retrograde root-end fillings and repair of lateral root perforations [5]. MTA characterized by low or no toxicity [6], shows excellent biocompatibility [7] and stimulates repair [8, 9], as it allows cellular adhesion, growth and proliferation on its surface [10]. MTA has good antibacterial properties but it doesn’t show important cytotoxicity effect on host cells [11]. In addition, it induces cementogenesis [12], while regenerating the periodontal ligament and leads to bone formation [12, 13].

In some clinical applications such as retrograde filling and guided tissue regeneration, an accelerated setting time is required to overcome dissolution of materials in oral fluids and blood. In spite of many outstanding mechanical and biological properties, MTA has several disadvantages such as difficult handling properties and long setting time [14, 15]. New CSCs like RetroMTA (BioMTA, Seoul, Korea) may overcome these problems [16].

Retro MTA is a fast setting CSC which consists of calcium zirconia, aluminum oxide, silicon dioxide, and calcium carbonate [17]. the manufacturer claimed the initial setting takes place only in 150 s [18] recently in an Invitro study Retro MTA showed significantly lower initial and final setting time compared to Angelus MTA and calcium-enriched mixture (CEM) cement [16]. Moreover Retro MTA showed a variety of favorable properties such as high biocompatibility and good cell viability [17, 19].

Minimal degradation and slow resorption of various types of MTA were reported in previous studies [11, 20, 21]. On the other hand, β-TCP is a biodegradable material and it is considered to be resorbed at a higher rate than tissue formation [22, 23].

Calcium phosphate [CaP] showed good effects when used as bone graft material. CaPs such as hydroxyapatite and β-tricalcium phosphate [β-TCP] are highly biocompatible and are generally accepted to be osteoconductive and bioactive when implanted into osseous defects [24, 25]. β-TCP can be considered as a scaffold for new bone formation and it may increase the ingrowth of capillaries, perivascular tissue and osteoprogenitor cells into the recipient site [26, 27].

As mentioned, Different biodegradation rates have been reported for Retro MTA and TCP. While β-TCP biodegrade in a rather rapid and unpredictable way [23, 28], Retro MTA may provide more stability [15, 16, 20, 21]. Based on our hypothesis a Retro MTA/ β-TCP mixture not only preserve the advantages of both materials but also can cover the shortcoming of their biodegradation rates by providing a scaffold with an averaged resorption time.

Hence, the objective of this pilot animal study was to evaluate the efficacy of a Retro MTA/ β-TCP mixture for periodontal tissue regeneration in dehiscence defect model.

## Material and methods

This animal study was approved by the local ethical committee of the Isfahan University of Medical Science (Registration Number: 3941015).All procedures in this experiment carried out in accordance with the National Institutes of Health guide for the care and use of Laboratory animals [29].

Four healthy Beagle dogs (12–36 months old, weighing 20 to 25 kg) were included in this project. Animals were anesthetized by injection of 2% acepromazine and 10% Ketamine Hcl. After an injection of 0.1% atropine (Atropine, alfasan, Woerden, Netherlands; 0.02–0.04 mg/Kg), dogs have been intubated and halothane gas (Halothane BP, Nicholas Piramal India Limited, India) was used to maintain the anesthesia. Local infiltration with lidocaine (persocaine-E,Lidocaine HCL 2% + Epinephrin1/80000, Daroupakhsh pharmaceutical. Mfg Co. Tehran, Iran) was used to control any pain and bleeding during the surgical procedure. Disinfection of the treated sites and the mouth was performed with 0.2% chlorhexidine solution before the surgery using swabs.

**Fig. 1:**
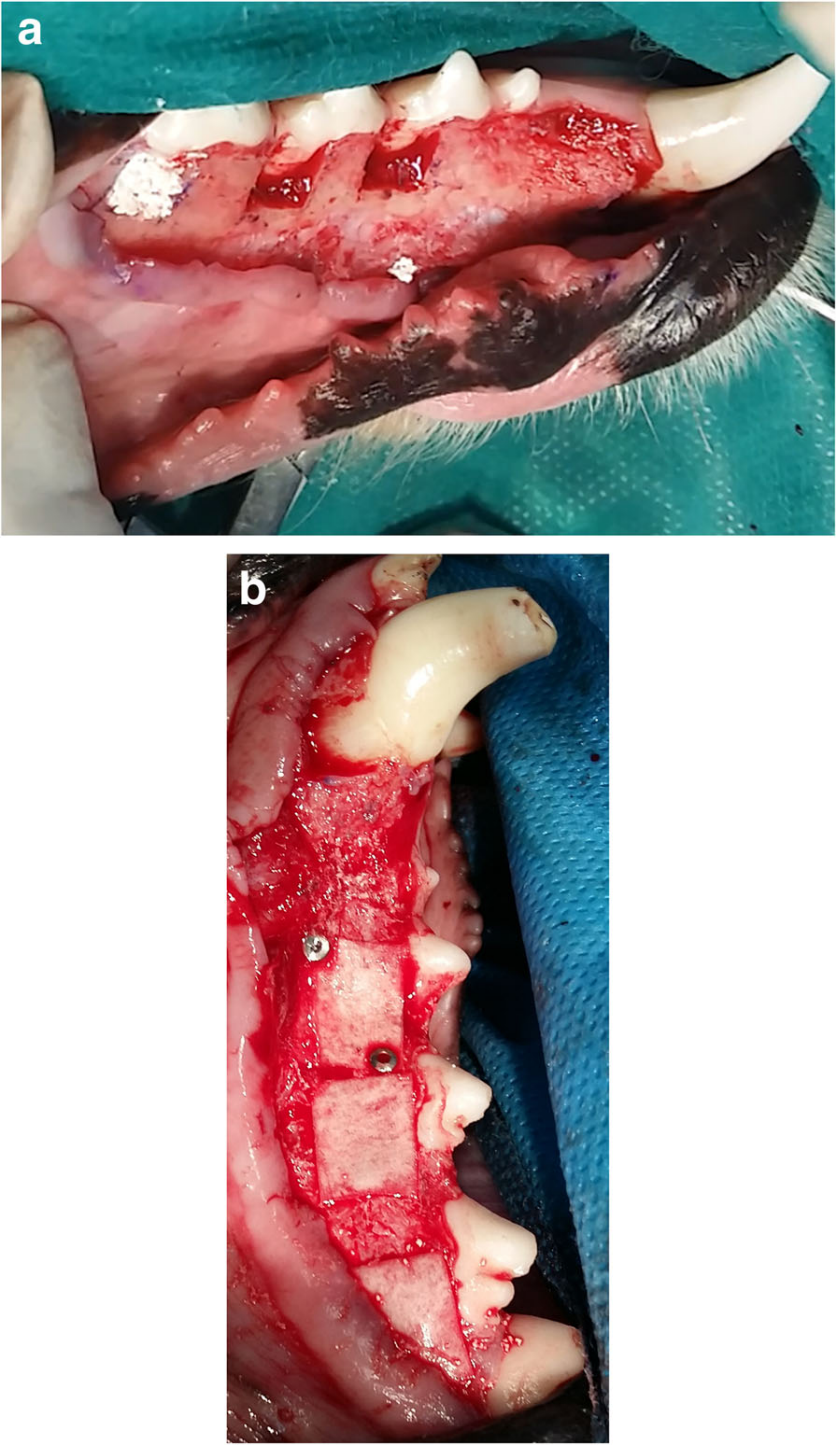
**a** Surgical Procedure of creating four standardized critical size periodontal dehiscence defects on each side of the mandible. One of the defects in this photo was filled with Retro MTA + TCP. **b** Surgical Procedure of treating the defects and fixing the collagen membranes with titanium pins

After sulcular incision from mandibular first molar to canine, a muco-periosteal flap was elevated using an elevator. A carbide bur, Gracey curettes and bone chisel were used to create four standardized critical size periodontal dehiscence defects on each side of the mandible, resulting in eight defects per dog. For this purpose 5 × 5 mm of the supporting bone were removed and the periodontal ligament including the cementum were removed from the roots of the canines and the distal roots of the 2nd, 3rd and 4th premolars (Fig. 1a). A 5 mm long notch was prepared at the apical end of each defect with half-round carbide bur (No. 2), which served as a reference point for preparation of histologic sections. Defects were rinsed with physiologic saline. On each side of the mandible, two defects were left unfilled and served as control sites, whereas the remaining two defects were randomly filled with Retro MTA or a mixture of Retro MTA + β-TCP (Kasios, Lanauguet, France). The mixture was achieved by admixing equal volume of Retro MTA powder and β-TCP. Retro MTA and Retro MTA/ β-TCP powder were separately mixed with the liquid (distilled water) based on the manufacturers’ guidelines under sterile conditions.

All test defects and one of the control defects were covered with a collagen membrane (Jason®, Botiss Biomaterials, Berlin, Germany) (Fig. 1b). Their placement was extended at least 2 mm beyond the defect borders and all membranes were stabilized and fixed with titanium pins (AutoTac System, Biohorizons, Birmingham, USA)The flaps were released, carefully repositioned and sutured using 0-3PTFE (Osteogenics Biomedical, Inc., USA).

These procedures were performed on both sides of each dog’s mandible. Thereby, a total of 32 periodontal dehiscence defects were created consisting of 16 control defects and 16 experimentally filled defects, which were covered with membranes. There were two control groups (each *N* = 8): Control group 1 was left unfilled and was just covered with a collagen membrane, whereas control group 2 was left without any treatment.

Post-surgical food & drug considerations have been planned for the animals as indicated in standard protocols [30]. Animals were sacrificed via intravenous injections of ketamine, magnesium sulfate and acepromazineat after 8 weeks.

Tissue blocks were prepared and placed in 10% formalin for 2 weeks. Afterwards, specimens were rinsed with physiologic saline for 10 min and placed into 10% formic acid at room temperature for decalcification. Specimens were then dehydrated in ascending concentrations of alcohol and were embedded in paraffin. From each defect, five 5-μm-thick sections were prepared and stained with hematoxylin and eosin (H&E). The sections were obtained from standardized different apico-coronal levels of each defect and the direction of sectioning was bucco-lingual. In each defect the most apically section was obtained from 1 mm level above the apical reference notch and the other sections prepared at even intervals of 0.75 mm.

All specimens were then histomorphometrically investigated by a blinded oral pathologist using an optical microscope (Nikon E400, Japan) at magnifications of × 100.

The following histomorphometric evaluations were made:

1. Thickness of new cementum,
2. Thickness of newly regenerated bone
3. Thickness of the periodontal ligament (PDL)

**Fig. 2:**
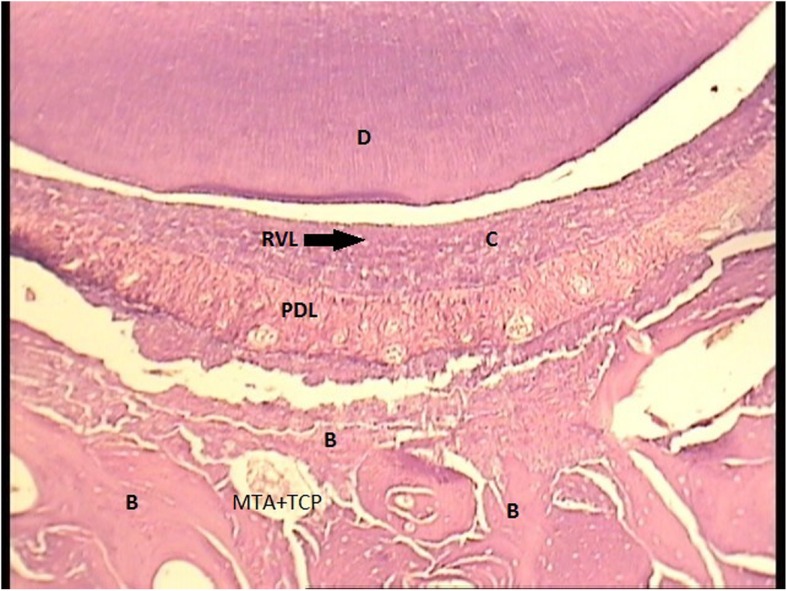
Histologic image of defect treated with Retro MTA + TCP (magnification × 100). C: Cementum B: new Bone D: Dentin RVL: Reversal Line

In a few number of histologic slides, an auxiliary criterion for distinguishing newly formed cementum/bone from probable pre-existed cementum/bone in microscopic evaluation was reversal line. In these cases, the hard tissues (cementum/bone) which have been formed during the 8 weeks were less calcified than the remained bone and cementum (Fig. 2).

**Fig. 3:**
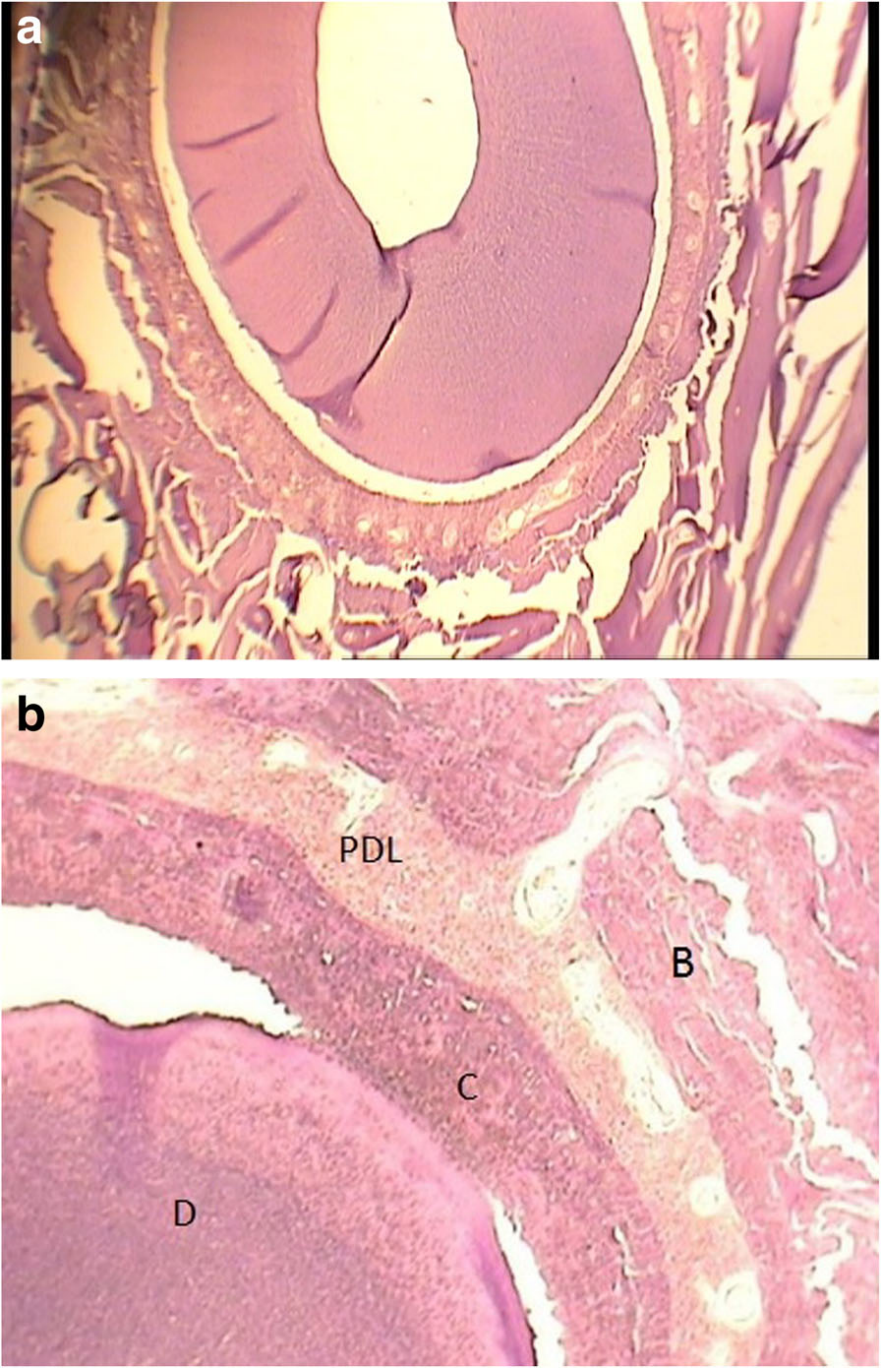
**a** Histologic image of No. 1 control group (magnification × 40). **b** Histologic image of No. 1 control group (magnification × 100)

In addition, an inflammatory score was assessed under the optical microscope based on the following classification [31]:

Score 0: < 10% inflammatory cells, Score 1: 10–30% inflammatory cells, Score 2: 30–50% inflammatory cells, Score 3: > 50% inflammatory cells.

The statistical analysis was performed with Kruskal-Wallis, Mann–Whitney and Fisher’s exact statistical tests (α = 0.05) using the SPSS software 16 package (SPSS™,SPSS Inc., Chicago, USA).

## Results

### Clinical observations

Healing occurred uneventfully in all animals. Only two defects in one dog showed slight wound dehiscence formation 2 days after surgery, which had to be sutured again. During the 2 months healing period, no visible adverse reactions, such as infection, suppuration or root exposure were observed. No significant inflammatory reactions were seen.

### Descriptive histological findings

Histological analysis could be performed for all defects. No signs of acute inflammation were observed in any of the defects.

Chronic inflammatory response was present in all specimens. All cases showed score 0 (less than < 10% inflammatory cells) regarding the inflammatory classification except for two defects which showed slight wound dehiscence during first week of post-surgical period. These two defects received score 1 (10–30% inflammatory cells) of the classification.

In defects filled with Retro MTA or Retro MTA + TCP, collagen fibers inserting in the newly formed cementum could be observed in all defects.

Periodontal ligament formation in control groups could also be observed in five defects of the membrane group and 4 defects of empty group.

**Fig. 4:**
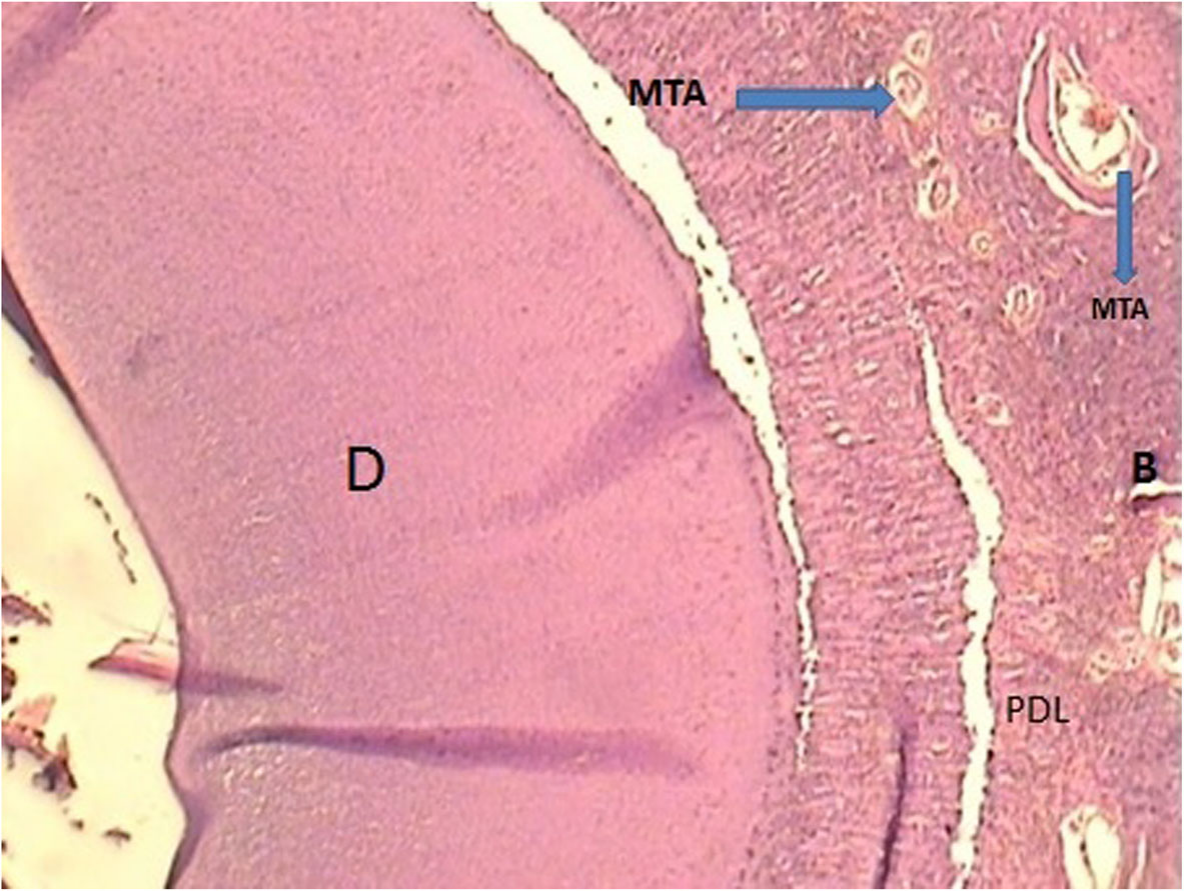
Histologic image of defect treated with MTA (magnification × 100)

**Fig. 5:**
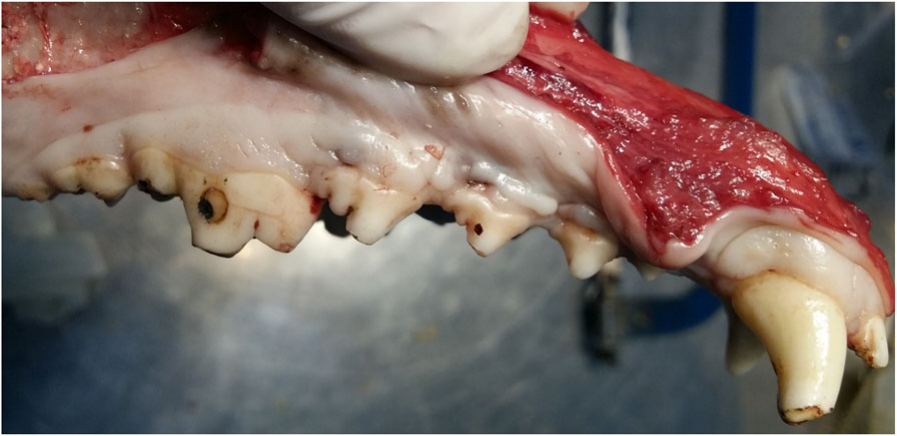
Clinical image of mandible at the end of 8th week. No sign of inflammation or adverse reaction can be seen in clinical view.

New cementum formation was observed in all defects of Retro MTA+ β-TCP group and 80% of defects in Retro MTA group (Figs. 2, 3, 4 and 5). In contrast, cementum formation was observed in 45% of the unfilled membrane group.

With regard to the formation of bone during the healing time, 100% bone formation was found in 5/8 defects treated with Retro MTA+ β-TCP and 4/8 defects where Retro MTA was applied. None of the control groups showed 100% bone formation.

### Histomorphometric analysis

Table 1 shows the average of measures which obtained from all 40 sections of 8 defects in each category.

**Table 1:** Histometric analyses of the periodontal tissue formation [mean ± SD, mm]

	MTA	MTA + TCP	NO 1 control ^¥^	NO 2 control^₤^
New bone	2.25 ± 0.24 *	2.87 ± 0.42 **	1.25 ± 0.32	0.55 ± 0.45
New cementum	3.24 ± 0.56 *	3.74 ± 0.34 **	1. 15 ± 0.45	0.78 ± 0.65
New PDL	4.32 ± 0.35	4.45 ± 0.25	3.14 ± 0.45	2.65 ± 0.67

*n* = 8 for each treatment

*Statistically significant difference between MTA group and both of control groups (*P* < 0.0001)

**Statistically significant difference between MTA + TCP group and both of control groups (*P* < 0.0001)

^¥^Control group 1: The defects which were left unfilled and just covered with a collagen membrane

^₤^Control group 2: The defects which were left without any treatment

Results showed that the formation of newly formed bone and cementum in the experimental groups was significantly higher as compared to the control groups (*P* < 0.0001). In addition, periodontal ligament formation was more accentuated in the experimental groups. However, no significant difference between the experimental and the control group could be observed (*P* = 0.92).

Although there was a tendency for more periodontal tissue regeneration in defects of Retro MTA+ β-TCP group as compared to the Retro MTA group, this was not statistically significant (*P* = 0.08).

## Discussion

Despite the progress made in application of MTA and the other CSCs in dental pulp regeneration and the endodontic procedures, data assessing the application of MTA regarding the regeneration of periodontal are still scarce if not inexistent. In the present study, MTA and β-TCP were used based on the hypothesis that they have a potential for periodontal tissue regeneration. Therefore, the main aim of this study was to evaluate and compare the efficacy of Retro MTA or a Retro MTA + β-TCP mixture for regeneration of surgically created periodontal dehiscence defects in dogs. The results showed that the use of Retro MTA or Retro MTA+ β-TCP in combination with a GTR technique could enhance the regeneration procedure of standardized dehiscence defects.

According to Wikesjö and Selvig, experimental defects commonly considered for regenerative therapy have included defects caused by natural periodontal disease or defects induced by plaque retaining crevicular ligatures (chronic model) [32]. As a limitation of the natural periodontal diseases, the extent and localization of periodontal lesions are not always synchronized in dogs [33]. Chronic defects are characterized by compromised periodontal dimensions which may represent a confounding factor in evaluation of the healing results. Therefore, Non-infection model was selected for this study.

Previous in vitro studies have shown that MTA may not only serve as an inert material but rather appears to be a material with distinct biological activity towards fibroblast proliferation and cementum formation [34–36]. Several clinical studies confirmed the successful regeneration of bone in defects treated by MTA and/or β-TCP [37–39]. A recent systematic review regarding different applications of MTA and other CSCs concluded that the evidence regarding the use of CSCs during periodontal treatment is limited [40].

A clinical study in humans used ProRoot MTA combined with a collagen membrane trying to treat the Class II furcation defects in mandibular molars [41]. Results after 6 months showed a significant decrease in probing depth and improvement of the horizontal and vertical clinical attachment levels of the experimental areas [41].

A potential limitation of MTA is its low solubility and in vivo resorption profile. While MTA provides a potential matrix for tissue ingrowth and ongrowth, the long-term presence can also eventually limit periodontal tissue formation. On the other hand, Beta-tricalcium phosphates showed increased biodegradation rates, however, in a more unpredictable way, as shown in different studies [28, 42]. The mixture of MTA and β-TCP may overcome such long-term limitations as compared to the separate use of each material per se, which was corroborated by the results of the present study, which showed better effect of Retro MTA plus β-TCP as compared to Retro MTA alone.

The first aim of this study was assessing the possibility of using Retro MTA and Retro MTA + β-TCP in periodontal regeneration process. It must be admitted that this analysis has some limitations and shortcomings. The method of choice in our study was based on measuring the thickness of regenerated tissues. It is necessary to conduct linear measurements in future studies to compare the results with other papers regarding regenerative techniques. Moreover, using advanced methods such as μ-CT scans would help to make a definitive decision in the future on the option of using these materials for treatment of periodontal defects.

## Conclusion

Retro MTA or Retro MTA+ β-TCP covered with a collagen membrane used in a dehiscence type defect resulted in regeneration of periodontal tissues. However, Retro MTA+ β-TCP showed tendency towards better results than the use of Retro MTA alone. The materials under investigation may have some potential to regenerate periodontal tissues and can be considered as a novel regenerative therapy in periodontics. However, more research is needed in other defect types and in long-term pre-clinical and clinical trials.

## Data Availability

The dataset supporting the conclusions of this article available and will be presented based on request.
